# Enhancing tuberculosis care in the private sector: Role of innovative private sector engagement model under programmatic settings in India

**DOI:** 10.1371/journal.pgph.0006333

**Published:** 2026-05-08

**Authors:** Sandhya Gupta, Raghuram Rao, Kathirvel Soundappan, Kedar Mehta, Suseendar Shanmugasundaram, Akash Ranjan Singh, Hitesh Verma, Alok Mathur, Rajendra Panduranga Joshi, Prabhadevi Ravichandran, Urvashi Balbir Singh

**Affiliations:** 1 Karnataka Health Promotion Trust, New Delhi, India; 2 Central TB Division, Ministry of Health and Family Welfare, New Delhi, India; 3 Department of Community Medicine and School of Public Health, Post Graduate Institute of Medical Education and Research, Chandigarh, India; 4 Dahdaleh Institute for Global Health Research, Faculty of Health, York University, Toronto, Canada; 5 Department of Community Medicine, GMERS Medical College, Gotri, Vadodara, Gujarat, India; 6 Department of Community Medicine and Family Medicine, All India Institute of Medical Sciences, Hyderabad, India; 7 Department of Community Medicine, Government Medical College, Shahdol, Madhya Pradesh, India; 8 Directorate General of Health Services, Health Department, Government of Haryana, Panchkula, India; 9 National Centre for Disease Control, Directorate General of Health Services, Ministry of Health and Family Welfare, New Delhi, India; 10 Directorate General of Health Services, Ministry of Health and Family Welfare, New Delhi, India; 11 Department of Community Medicine, Chettinad Hospital and Research Institute, Chettinad Academy of Research and Education, Kelambakkam, TamilNadu, India; 12 Tuberculosis Division and Department of Microbiology, All India Institute of Medical Sciences, New Delhi, India; Dr D Y Patil Medical College Hospital and Research Centre, INDIA

## Abstract

India’s private sector plays a crucial role in the country’s tuberculosis (TB) healthcare landscape, with >50% of patients seeking initial care from private providers. Recognizing this critical role, the National Tuberculosis Elimination Program implemented and scaled up an innovative private sector engagement model, Patient Provider Support Agency (PPSA), in over 200 districts of the country in 2023 to improve TB care in the private sector. A cross-sectional study was conducted to assess the role of PPSA in improving TB care services among private-sector patients in 2023. We compared districts with PPSA and without PPSA for their TB notification, treatment timeliness, key quality-of-care indicators and treatment outcomes from the private sector at the national level. Data for private sector notification from *Ni-kshay* (web-enabled TB patient management system) was analyzed. In 2023, districts supported by PPSA (n = 204) recorded an average private sector TB notification rate of 106 per 100,000 and achieved 99.7% of the set target, compared to 529 districts without PPSA, which reported a rate of 45 per 100,000 and reached only 80% of the target. The pretreatment loss to follow-up (2% vs 4%) and the treatment initiation delay >7 days (5% vs 10%) were lower in districts with PPSA. They also reported improved TB care services- comorbidity testing (HIV and diabetes), bank detail linkage for receiving financial support and treatment success with PPSA support. However, districts with PPSA showed lower coverage of upfront nucleic acid amplification testing (17% vs 22%), drug susceptibility testing (21% vs 25%) and uptake of government-supplied drugs (22% vs 33%) compared with districts without PPSA. The PPSA model improved private sector TB notification and reduced treatment delays nationally; however, strategic expansion by focusing on quality-oriented TB care services and their link to results-based financing is essential to maximize its efficiency and overall impact on private sector TB care.

## Introduction

India shares the highest burden of tuberculosis (TB) with 26% (2.8 million) estimated global TB incidence cases and 28% (0.3 million) global deaths in 2023 [1]. Of the estimated cases, 2.5 million cases were notified in the same year – the highest-ever TB notification in India. The private sector accounted for approximately one-third of the total notifications [2].

Evidence suggests that the private sector often serves as the initial point of care for 50–70% of TB patients. On average, individuals with TB or respiratory symptoms consult three healthcare providers (predominantly private providers) before undergoing the appropriate diagnostic evaluation [3,4]. Considering the extensive presence and influence of the private sector within the country’s TB healthcare landscape, its engagement remains a pivotal focus for the National Tuberculosis Elimination Programme (NTEP) [5].

To strengthen private sector engagement, NTEP has implemented several public–private mix (PPM) approaches over the past decade [5,6]. Early initiatives, including grant-in-aid mechanisms and partnerships with non-governmental organizations, improved access to TB services but were often limited by rigid design, ad-hoc implementation, contextual relevance and insufficient patient-centricity [6,7]. Over the years, private providers contribution to TB diagnosis and treatment improved, however challenges persisted in ensuring timely notification, adherence to national diagnostic algorithms, and delivery of standardized, high-quality TB care [7–10]. Transitioning from traditional PPM strategies, in 2014 NTEP piloted the innovative “Private Provider Interface Agency” (PPIA) model with donor support in Patna, Mumbai and Mehsana of India [7]. With the involvement of PPIA, the annual TB case notifications in Mumbai rose by 58%, and the TB notification rate almost doubled between 2013 and 2017 [11]. Further, a lower diagnostic and treatment delay was reported among patients from the private sector linked with PPIA compared to patients without linkage with PPIA.[12] However, the PPIA model was resource intensive and largely donor supported, raising questions about its scalability within routine programme settings [7,13].

Based on the lessons learned from the PPIA pilot, the Patient Provider Support Agency (PPSA) was implemented in 105 districts, a relatively less resource-intensive model with funding from the Global Fund (from 2018 to 2020) [7]. PPSA functions as an intermediary between private providers and the public health system, supporting TB notification, linkage to diagnostics and treatment, patient follow-up, comorbidity screening, and access to programmatic benefits [6,14,15]. Although PPSA have demonstrated significant improvements in private sector TB notification, it was predominantly donor-driven with rigid operational designs, restricting adaptations to local epidemiological and health systems contexts. Importantly, the sustainability of the intervention and its effects have been identified as a crucial issue [7]. Hence, NTEP has integrated and scaled up this PPSA intervention to over 200 districts across India by 2023 using domestic funding [2,15].

Although few studies have evaluated the initial PPIA/PPSA implementation in different parts of the country, these were limited to a) implementation in selected small study locations; b) comparison of only pre and post-PPSA implementation assessment, rather than comparing between PPSA and non-PPSA districts; and c) assessing private sector engagement models run under donor funding sources rather than domestic funding. [11,12,16]

Hence, we conducted this analysis aimed to assess the impact of domestically funded PPSA implementation on private-sector TB notifications, treatment timeliness, outcomes and key quality-of-care indicators—including nucleic acid amplification testing (NAAT) testing, comorbidity screening, and access to programmatic benefits, by comparing districts with and without PPSA support under routine programmatic settings in India.

## Methodology

### Study design

This was an analytical cross-sectional study based on secondary programmatic data analysis at the national level.

## Setting

### General setting

The NTEP in India operates under a well-defined structure extending from the national to the peripheral level to ensure comprehensive TB care services. At the national level, the programme is led by the Central TB Division (CTD). State and district-level program planning, implementation, and supervision are coordinated by 36 State TB Cells (one in each State or Union Territory) and 771 District TB Centres (at each district), respectively [17]. At the sub-district level, Tuberculosis Units (aligned to the blocks), provide decentralized services for TB diagnosis, treatment, and care. The smallest operational units under NTEP are the Peripheral Health Institutions (PHIs), which include public or private facilities staffed by medical doctors. These facilities may be equipped with sputum microscopy or NAAT machines, functioning as TB Diagnostic Centres (TDCs) and ensuring accessible diagnostic, treatment and referral support.

To enhance private sector TB case detection, the NTEP advocates adherence to national standards of TB care [18] and mandates all private practitioners (as per Gazette notification 2012) to notify every TB case through the *Ni-kshay* portal (web-based patient management information system for TB). NTEP has incentive-based enablers to improve TB notification and outcome reporting from private providers. In routine program settings, State and district level NTEP staff- Senior Treatment Supervisors (STS) and Public-Private Mix (PPM) coordinators are responsible for engaging and monitoring private healthcare providers for TB notification and treatment outcomes.

In 2023, India reported the highest private sector TB notification ever, accounting for 33% (0.84 million) of total notifications. There is a notification gain of 15% over last year from the private sector, while the public sector notification improved by 1% only [2,14].

### Specific setting

**Fig 1:**
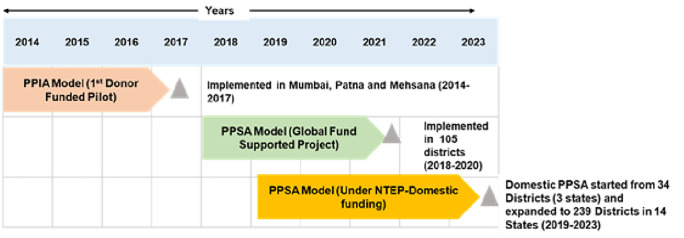
Patient Provider Support Agency (PPSA) model evolution and scale up in India. *PPIA (Public Private Interphase Agency); PPSA (Patient Provider Support Agency); NTEP (National Tuberculosis Elimination Programme)*.

**Fig 2:**
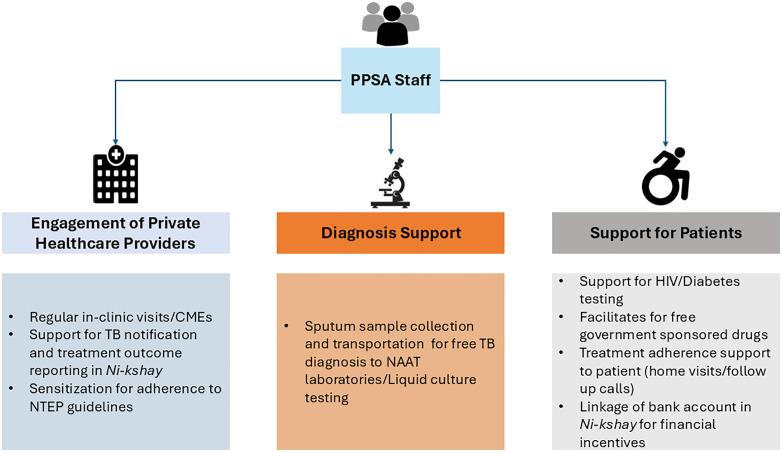
TB care services supported through PPSA model at district level under NTEP, India. *PPSA (Patient Provider Support Agency); CME (Continued Medical Education); NAAT (Nucleic acid amplification test); NTEP (National TB Elimination Programme)*.

Given the vast number of private healthcare providers and limited programmatic field staff, for effective engagement of the private sector [5], NTEP has implemented an innovative private sector engagement model known as the Patient Provider Support Agency (PPSA). This model evolved through successful donor-funded pilot projects (Fig 1) [7]. PPSA act as an intermediary between NTEP and private healthcare providers to offer comprehensive TB services for private sector patients including mapping and landscaping of private providers, sensitizing private providers to ensure TB notification and outcome reporting, linkage to TB care services like free diagnostics and treatment services, patient counseling and adherence support, comorbidity screening, drug susceptibility testing (DST) through NAAT and support for accessing financial benefits under programme (Fig 2) [6,14].

The deployment of the PPSA model is characterized by significant variation across Indian states, as health is a state subject and is tailored to address local needs and contextual requirements. State and district health authorities conduct systematic assessments to identify critical gaps in TB service delivery. These include deficiencies in diagnostic capacity, logistical infrastructure, and human resources, as well as performance indicators such as low rates of TB notification and service provision by private healthcare providers. Identified gaps are triangulated with state/district-level data sources—such as private sector drug sales, registries of private healthcare providers, the proportion of providers reporting through Nikshay, and existing infrastructure and workforce capacity to evaluate the extent of private sector underperformance and to inform the scope and nature of required partnerships [15].

The prioritization of districts or states for PPSA implementation is influenced by a complex interplay of factors. These include the high density of private healthcare providers, gap between private notification and TB burden, the existence of alternative models for private sector engagement, administrative readiness, budgetary allocations, and the institutional capacity to manage PPSA contracting processes [5,15].

To operationalize the PPSA model, eligible entities usually non-governmental organizations (NGOs) are selected through a competitive tendering process led by state or district authorities. These NGOs are financed through domestic resources under a results-based financing framework, which emphasizes efficiency and accountability. Fund disbursement is done based on the verification of achieved predefined targets related to TB notification, treatment outcomes, and the delivery of essential TB care services [15]. (Refer operational definitions in S1 Text).

### Study site

**Fig 3:**
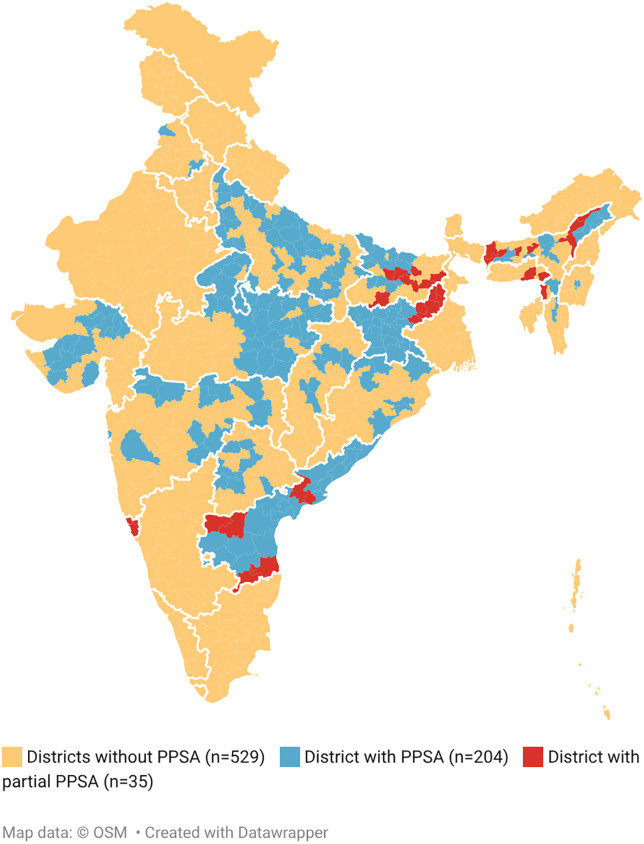
Map of PPSA coverage across all States/Union Territories of India during 2023. This map was generated using openly available basemap sourced from Datawrapper. The district boundaries were overlaid with programmatic data on PPSA implementation for 2023.

This was a national-level study to assess the effect of PPSA using existing programmatic data. Out of all the 771 NTEP districts, the study included 204 districts (from 14 States) with PPSA and 529 districts without PPSA support (from all 32 States/Union Territories) across India (Fig 3) for comparing their performance on private sector TB notification and care parameters, including treatment outcomes in 2023. The districts which had the presence of functional PPSA throughout the year during 2023 were considered as districts with PPSA support. The 35 districts that had PPSA presence for only part of 2023 were excluded. (Refer list of states and districts with PPSA operations at S1 Table and without PPSA operations at S2 Table, included in the study and districts excluded at S3 Table). As described above, the selection of districts for PPSA implementation was based on programmatic considerations that varied across Indian states; consequently, this process may have introduced systematic differences between PPSA and non-PPSA districts due to selection effects and underlying differences in baseline characteristics.

### Study population

The district served as the unit of analysis for the study. All people with TB (PwTB) notified from the private sector in selected districts from January to December 2023 were included. Private sector TB cases for whom the diagnosing district (from where the case gets notified) and current district (where the patient initiates treatment or gets follow-up services) remained different, were excluded as PPSA may not offer its complete services efficiently to such ‘transferred-in’ or ‘transferred-out’ patients.

### Data variables

The study analyzed secondary programmatic data obtained from *Ni-kshay*. The primary variables included were (i) private-sector TB notification against the target set under NTEP (State and district wise targets for private sector TB notifications under NTEP are derived from estimated TB burden, past notification trends, and private healthcare presence annually by them) and the notification rate (notification against per lakh population) in districts with and without PPSA from January to December 2023 (ii) Demographic, behavioral, and clinical characteristics of notified PwTB, such as age, gender, tobacco use, alcohol consumption, type of TB based on site of disease, drug susceptibility, previous TB history and basis of diagnosis were assessed. (iii) Similarly, pre-treatment loss-to-follow-up (LFU) among notified cases (includes all those who did not initiate treatment after being notified for TB), delays of more than seven days in treatment initiation were calculated using the dates of diagnosis and treatment initiation. Under the NTEP treatment should be initiated *immediately* after diagnosis—ideally on the *same day*—to reduce transmission and improve outcomes. For the study, delay of more than seven days was considered as a delay in treatment initiation. (iv) Efforts to offer diagnostic tests were assessed by comparing the proportion of bacteriological confirmation and upfront NAAT. (v) Furthermore, TB care services offered to patients who started on TB treatment were analyzed. This included valid DST, HIV and diabetes testing, provision of free government fixed-dose combination (FDC) drug, linkage of bank account details with *Ni-kshay* for financial support to address the nutritional needs of patients under *Ni-kshay* Poshan Yojana (NPY) and treatment duration. (vi) Lastly, treatment outcomes- cure, success, loss to follow-up, death, or non-evaluation were also analyzed.

### Data analysis

The secondary programmatic data (without patient identifiers) on TB notification, pre-treatment loss-to-follow-up, pre-treatment delay, receipt of public health actions, and treatment outcomes covering the period January-December 2023 for PPSA and non-PPSA districts were extracted from *Ni-kshay* in December 2024. We extracted the data in December 2024 to enable the complete reporting of the treatment outcomes for all PwTB who were notified during the study period. The list of districts with and without PPSA operations in 2023 retrieved from routine program records, was merged with the *Ni-kshay* database using the diagnosing district name.

All variables were summarized descriptively and compared between PPSA and non-PPSA districts. Categorical variables were presented as proportions. Absolute differences between PPSA and non-PPSA districts were calculated along with their corresponding 95% CIs to complement percentage comparisons.

The difference in TB notifications, their demographic, behavioral, clinical characteristics and TB care parameters were tested using chi-square test (for categorical variables) among PPSA and non-PPSA districts. The p-value < 0.05 was considered statistically significant. The analysis was done using Microsoft Excel soft Excel 2021 MSO (Version 2509).

### Ethics approval

Ethics approval was obtained from the Institutional Ethics Committee of the Indian Council of Medical Research-National Institute of Epidemiology (ICMR-NIE), Chennai (NIE/IHEC/A/202408–05 dated 11^th^ September 2024). As the study involved an analysis of secondary data, a waiver for informed consent was obtained from the ethics committee.

## Results

In 2023, districts with PPSA (n = 204) and without PPSA (n = 529) notified a total of 445,558 and 341,398 TB cases from the private sector, respectively. This is equivalent to an average TB notification rate (NR) of 106 per 100,000 population in districts with PPSA in comparison to 45 per 100,000 in districts without PPSA. The private sector TB notification of 99.7% was achieved against the target (n = 446,755) set for PPSA districts under NTEP. In contrast, districts without PPSA reported 80% notification achievement against the set target (n = 426,574).

### Demographic, behavioural and clinical characteristics of PwTB notified from the private sector in 2023

Most PwTB reported were individuals aged 15–44 in both district groups. Similarly, gender distribution showed a male predominance in both district groups. Although tobacco (3% vs. 6%) and alcohol (1% vs. 4%) use were reported in PPSA and non-PPSA districts, the proportion of “Not Recorded” entries was substantial—97% vs. 94% for tobacco use and 41% vs. 23% for alcohol use. The proportion of pulmonary TB (PTB) cases was higher in districts with PPSA, while extra-pulmonary TB cases were higher in those without PPSA. The distribution of drug-sensitive tuberculosis (DSTB) and drug-resistant tuberculosis (DRTB) was comparable in both groups. A lower bacteriological confirmation (26% vs 32%) and upfront NAAT testing (17% vs 22%) were found in districts with PPSA when compared with districts without PPSA (Table 1).

**Table 1:** Demographic, behavioral and clinical characteristics of PwTB notified from Private sector in the districts with and without Patient Provider Support Agency (PPSA) during Jan-Dec 2023, India.

Parameters	Districts		Districts		Absolute difference (95% CI)^
	**With PPSA (n-204)**		**Without PPSA (n-529)**	
	*N = 4,455,58	(%)	*N = 3,413,98	(%)	
**Demographic and behavioral characteristics**					
Age group (years)					
<15	35,871	8	22,763	7	1.4 (1.3 to 1.5)
15-44	2,50,083	56	1,83,695	54	2.3 (2.1 - 2.6)
45-64	1,13,034	25	92,489	27	-1.7 (-1.9 to -1.5)
≥65	46,546	10	42,424	12	-2 (-2.1 to -1.8)
NR	24	(<1)	27	(<1)	0
Gender					
Male	2,51,216	56	1,95,188	57	-0.8 (-1.0 to -0.6)
Female	1,94,190	44	1,46,052	43	0.8 (0.6 to 1)
Transgender	142	(<1)	152	(<1)	0
NR	10	(<1)	6	(<1)	0
Tobacco Users					
Yes	12,343	3	19,701	6	−3.0 (−3.1 to −2.9)
NR	4,33,215	97	3,21,697	94	3.00 (2.91 - 3.09)
Alcohol User					
Yes	5,375	1	12,386	4	-2.4 (-2.5 to -2.4)
No	2,57,115	58	2,51,093	74	-15.8 (-16.1 to -15.6)
NR	1,83,068	41	77,919	23	18.3 (18.1 -18.5)
**Clinical characteristics**					
Site of TB					
PTB	2,97,219	67	2,04,329	60	6.9 (6.7 - 7.1)
EPTB	1,39,851	32	1,25,022	37	−5.2 (−5.4 to −5.0)
NR	8,488	2	12,047	4	−1.6 (−1.7 to −1.5)
Drug susceptibility of TB					
DSTB	4,38,248	98	3,35,327	98	0.2 (0.1 -0.2)
DRTB	7,310	2	6,071	2	-0.2 (-0.2 to -0.1)
Type of TB cases					
New	4,16,466	93	3,04,294	89	4.3 (4.2 - 4.5)
Retreatment recurrent	4,114	1	5,093	1	−0.6 (−0.6 to −0.5)
Retreatment others	9,949	2	13,375	4	-1.7 (-1.8 to -1.6)
Incorrect entries	5,326	1	4,931	1	−0.2 (−0.3 to −0.2)
NR	9,703	2	13,705	4	−1.8 (−1.9 to −1.7)
Bacteriological Confirmation					
Present	1,16,820	26	1,10,816	32	−6.2 (−6.4 to −6.0)
Absent	3,28,738	74	2,30,582	68	6.2 (6.0-6.4)
Upfront NAAT testing					
Offered	75,504	17	73,914	22	−4.7 (−4.9 to −4.5)
Not Offered	3,70,054	83	2,67,484	78	4.7 (4.5- 4.9)

*# All parameters amongst districts with and without PPSA were significantly different with p value <*0.*0001, however, the magnitude of absolute differences varied across indicators implying clinical and programmatic relevance; ^* Units are in percentage points; *N-number of Private sector TB notifications, % -Column percentage; CI-Confidence Interval; PwTB- People with TB; PPSA- Patient provider support agency (interface agency to link private health facilities, their patients to NTEP for notification and quality TB care), NR- Not recorded, PTB – pulmonary tuberculosis; EPTB – extrapulmonary tuberculosis; DSTB-Drug Sensitive TB, DRTB-Drug Resistance TB*; NAAT-Nucleic acid amplification test;upfront NAAT- NAAT test is offered as first test to diagnose presumptive TB sample*

### Pretreatment LFU and treatment initiation delay

Districts with PPSA reported lower pretreatment LFU 2% (8577) among notified TB cases in comparison to 3.7% (12755) in districts without PPSA. Among TB cases initiated on treatment (436,981), a delay of more than 7 days in initiating treatment was witnessed for 5% of cases in PPSA districts in contrast to districts without PPSA where 10% delay was observed among 328,821 on-treatment cases (Table 2).

**Table 2:** TB notification, Care services and Treatment outcome among Private sector PwTB in districts with and without Patient Provider Support Agency (PPSA) during Jan-Dec 2023, India.

Parameters	Districts		Districts		Absolute difference (95% CI)^
	**With PPSA (n-204)**		**Without PPSA (n-529)**	
	**N**	**(%)**	**N**	**(%)**
**Private TB Notification against target**	4,45,558 / 4,46,755	100	3,41,398/ 4,26,574	80	19.7 (19.6 to 19.8)
Pre-treatment LFU (among TB notified)	8,577	2	12,577	4	−1.8 (−1.8 to −1.7)
Pre-treatment delay (>7 days) among initiated on treatment	23,607	5	32,973	10	-4.6 (-4.8 to -4.5)
**TB care cascade services**					
Valid drug susceptibility test among notified TB cases	**n = 4,45,558**	**(%)**	**n = 3,41,398**	**(%)**	
Available	94,480	21	84,666	25	−3.6 (−3.8 to −3.4)
Not available	3,51,078	79	2,56,732	75	3.6 (3.4 - 3.8)
					
**PwTB initiated on Treatment**	**n = 4,36,981**	**(%)/IQR**	**n = 3,28,821**	**(%)/IQR**	
HIV testing done					
Yes	4,23,070	97	3,15,479	96	0.9 (0.8–1.0)
No	13,911	3	13,342	4	−0.9 (−1.0 to −0.8)
Diabetes testing done					
Yes	4,19,224	96	3,07,340	93	2.5 (2.4–2.6)
No	17,757	4	21,581	7	−2.5 (−2.6 to −2.4)
Provided Government FDC					
Yes	95,002	22	1,07,200	33	-10.9 (-11.1 to -10.7)
No	3,41,979	78	2,21,621	67	10.9 (10.7 - 11.1)
					
Bank details seeding for NPY benefit supported	3,72,389	85	2,69,942	82	3.1 (3.0 - 3.3)
Median treatment duration (in days)	167	(167-168)	167	(167-167)	
**Treatment Outcome**					
Treated successfully	3,98,999	91	2,97,026	90	1.0 (0.9 -1.1)
Died	12,841	3	9,059	3	0.1 (0.0 - 0.2)
LFU	8,071	2	5,582	2	0.1 (0.1- 0.2)
Not evaluated	2,929	1	3,378	1	−0.3 (−0.4 to −0.3)
Treatment Failure	1,538	0	1,331	0	0
Others	4,770	1	3,771	1	0
NR	7,833	2	8,674	3	−0.8 (−0.9 to −0.8)

*## All parameters amongst districts with and without PPSA were significantly different with p value <*0.*0001, however, the magnitude of absolute differences varied across indicators implying clinical and programmatic relevance; ^* Units are in percentage points; *N-values in number, % -Column percentage; CI-Confidence Interval, PwTB- People with TB, PPSA- Patient provider support agency (interface agency to link private health facilities,* *their patients to NTEP for notification and quality TB care), FDC- Fixed dose combination; TB – tuberculosis; HIV – human immunodeficiency virus; DM- Diabetes Mellitus, NPY- Ni-kshay poshan Yojana, LFU-Lost to follow up; IQR- Interquartile range; Treated successfully-include treatment completed and cured; Others- include duplicate record, treatment regimen changed, untraceable migrant, wrongly diagnosed, NR- Not recorded*

### TB care services among private sector PwTB during 2023

A comparatively lower proportion of notified PwTB underwent valid DST in districts with PPSA (22%) than without PPSA (25%). The coverage of other TB care cascade services - HIV testing, diabetes testing, initiation on government FDC and bank detail seeding were 97%, 96%, 22% and 85% respectively in districts with PPSA in contrast to districts without PPSA (96%, 93%, 33% and 82% respectively). There was no difference reported in median treatment duration (167 days) across both set of districts.

Successful treatment outcomes were observed in 91% of cases initiated on treatment in PPSA-supported districts and 90% in non-PPSA districts with small absolute difference of 1 percentage point which indicates limited clinical relevance despite statistical significance (Table 2).

## Discussion

This is the first country-level analysis to report the effect of PPSA in improving private sector TB notification, care cascade services and treatment outcomes under programmatic settings in India.

### TB notification, Pretreatment LFU and Treatment initiation delays

The study highlighted the effectiveness of the PPSA model in enhancing private sector TB notifications with 2.4 times the increase in average TB notification rate in districts with PPSA compared to those without PPSA. Furthermore, the districts with PPSA achieved a significantly higher percentage of private sector TB notifications against annual targets set under the programme (99.7% vs. 80%). This reflects the strategic focus on prioritizing regions with a higher density of private healthcare providers for PPSA operationalization and its efforts in mapping and engaging the vast categories of private providers and their linkage under NTEP. A Mumbai-based study on a similar model, i.e., PPIA reported remarkable improvement in the overall and private sector TB notification during three and a half years of pilot implementation [11]. However, the later PPIA model seemed to be a more resource-intensive intervention compared to the PPSA model, anecdotally [7].

The study also observed a notable reduction in pretreatment LFU and treatment initiation delays in PPSA districts while comparing districts without PPSA. This also reflects PPSA’s role in sensitizing private providers on the importance of timely treatment initiation and regular follow up of notified PwTB by PPSA staff- through home visits or telephonic follow up. One of the previous studies assessing patient pathway delays also reported reduction in the time taken for the diagnostic care cascade after two years of implementation of the PPIA model [12].

### TB care services

In terms of TB care services, PPSA districts demonstrated better performance in comorbidity testing (HIV and diabetes), as well as bank detail linkage for accessing NPY benefits, compared to non-PPSA districts. The improvement in HIV testing was not as appreciable as in diabetes testing and bank detail seeding. This may be due to the maturity of TB-HIV collaborative activity under the programme, which got prioritized from 2009 onwards [19], where better awareness of cross-testing of HIV and TB, easy access to HIV kits, and regular progress review have enhanced HIV testing coverage across all districts. While the emphasis on TB-diabetes cross-testing and collaboration with non-communicable diseases (NCD) is in the nascent stage under the programme [20] Evidence suggests that private providers’ concerns regarding patient confidentiality, sensitive nature of HIV-related discussions, and the potential loss of patients from their facilities may contribute to lower uptake of HIV testing [21,22].

The study identified lower upfront NAAT and valid DST testing in PPSA-supported districts compared to districts without PPSA during the study period. There may be several plausible explanations for these findings. Firstly, it could be due to ineffective provider orientation on the latest NTEP diagnostic algorithms and UDST [5], which is reflected by absence of bacteriological confirmation among 74% cases. The greater reliance on clinical diagnosis in the private sector often results in lower rates of bacteriological testing, including NAAT [23–26]. Second, though PPSA actively provides sample collection and transportation support for NAAT testing at government laboratories, often systemic issues like inconsistent availability of NAAT machines/ cartridges/chips, shortage of laboratory personnel and delays in reporting test results, further discourage providers from recommending these tests. Previous private sector engagement models have demonstrated higher NAAT coverage when uninterrupted access was ensured through empanelment of private laboratories using project funds [11,12,27].

The study also revealed gaps in the proportion of private sector PwTB put on government FDC in PPSA-supported districts compared to their counterparts in non-PPSA districts. This discrepancy may be attributed to the private providers’ reluctance to prescribe government FDCs, especially the newly notifying providers. It may stem from various factors, including misconception that FDCs are intended for patients from lower income group, concerns about the quality of FDC drugs, fear of losing clients and autonomy, inadequate supply of FDCs under the program, patient reluctance and poor coordination between PPSA and district NTEP teams [28,29]. The low FDC coverage in PPSA districts also indirectly indicates the limited coverage or effectiveness of private providers engagement activities (trainings and sensitization) beyond case notification. Additionally, sustained trust-building efforts may be required to influence the prescription behavior of the private providers.

Furthermore, many PPSA contracts and performance matrices fail to integrate these quality care services into their payment structures, unlike notification and treatment outcomes reporting and hence tend to receive lower programmatic priority within the PPSA model [15]. This reflects systemic limitations of PPSA model and has implications for quality care services.

### Treatment outcomes

In 2023, treatment success rate approached 90% and was largely comparable between PPSA and non-PPSA districts, while other outcomes, including death, LFU, and treatment failure, remained mostly unchanged. This indicates improvements in routine program monitoring and outcome reporting across districts, independent of PPSA implementation. These findings suggest that while PPSA activities focus on timely initiation of treatment, patient follow-up and adherence support, they did not translate into measurable additional gains in treatment outcomes beyond those achieved through existing programmatic efforts.

## Strengths and limitations of the study

This study was conducted in a programmatic setting at the national level to assess improvement in private sector TB notification and TB care parameters on account of the PPSA engaged through domestic funding. This study stands apart from earlier smaller-scale studies in its broader scope and coverage.

Despite its strengths, the study has a few limitations. First, the districts compared in 2023 (those with and without PPSA) were diverse in terms of population size, TB burden, availability of healthcare resources, density of private providers and other contextual factors, which may affect their comparability. However, PPSA assignments have happened in a decentralized manner, and the investigators did not play any role in it. In addition, the analysis did not adjust for potential above-mentioned confounders, which may have influenced both PPSA implementation and observed outcomes.

The study employed a cross-sectional design using routine programmatic data of one year, which limits causal inference and precludes assessment of temporal changes attributable to PPSA implementation. As a result, observed differences between PPSA and non-PPSA districts should be interpreted with caution. Second, the comparison focused solely on private sector TB notifications without factoring in the corresponding public sector notifications during 2023 in the study districts. Adjusting for public sector TB notification rates would have provided the net improvement attributable to PPSA in the private sector. Third, the study did not evaluate PPSA’s role in mapping and engaging private health facilities. Analyzing the number of additional private healthcare providers mapped and registered on *Ni-kshay*, as well as the proportional increase in notifications from these facilities, would have offered valuable insights into the reach and effectiveness of PPSA in engaging diverse private sector providers. Fourth, the statistical significance is of limited clinical relevance, reflecting a small absolute difference amplified by the large dataset size. Lastly, the implemented PPSA intervention component may not be uniform across the district/state since they have been empowered to customize according to their local needs. Previous cost-effective analyses based on modelling inform the need to prioritize the various components within the PPSA interventions according to the local TB landscape [13,30]. Finally, a notable data documentation gap related to high proportion of “Not Recorded” entries for behavioral risk factors such as tobacco and alcohol use [31]. As incomplete recording constrains our ability to fully assess the role of PPSA in addressing behavioral risk profiling within the private sector, strengthening routine documentation of these variables may enhance the utility of programmatic data for future evaluations.

### Implications of the study & recommendation

The PPSA- model has significantly enhanced private-sector TB notifications across India, along with improving timely treatment initiation and TB care cascade services such as comorbidity testing and bank detail seeding for NPY. However, the study highlights a need to strengthen PPSA’s focus on comprehensive, quality-driven TB care, including services like upfront NAAT, DST, bacteriological confirmation, and promoting the adoption of government FDCs. Hence, a strategic shift is necessary—engaging private providers to deliver holistic TB care beyond notification. A performance matrix integrating all the above quality indicators, combined with a results-based financing approach, could potentially improve the efficiency of the programme [6]. Furthermore, it would strengthen private sector engagement activities and processes, which may lead to sustained long-term benefits including ensuring quality of care. Future studies exploring the facilitators and challenges of PPSA implementation, incorporating perspectives of PPSA agencies and private providers, and evaluating the different types of performance metrics and incremental costs of the PPSA model under NTEP would generate important evidence to guide refinement, scale-up and sustainability of the model. Since PPSA was implemented at different times across districts with varying contextual factors, comparing pre- and post-PPSA implementation outcomes is crucial to understanding the observed changes.

## Conclusion

This nationwide study found that the PPSA model has been successful in strengthening private sector engagement under NTEP, as reflected by higher TB notification rates, reduced pretreatment loss-to- follow-up, and shorter delays in treatment initiation in PPSA-supported districts. However, these improvements were not accompanied by proportionate gains in comprehensive quality-driven TB care like coverage of upfront NAAT, DST, bacteriological confirmation, and uptake of government-provided FDC in PPSA districts. To enhance the effectiveness of PPSA, its future implementation under NTEP should move beyond notification-focused performance metrics and strategically mandate the integration of TB quality care indicators into results-based financing contracts for all PPSA tenders. Systemic training of private providers on national diagnostic algorithms, access to NAAT services and improved laboratory capacity with faster turnaround under program are also critical to ensure provider engagement under the model.

## Supporting information

S1 Text Operational Definitions. (DOCX)

S1 Table List of states and districts with PPSA operations included in study. (DOCX)

S2 Table List of states and districts without PPSA operations included in study. (DOCX)

S3 Table List of states and districts excluded from the study. (DOCX)
