# Supplementary material for: Enhancing tuberculosis care in the private sector: Role of innovative private sector engagement model under programmatic settings in India
Source: PLOS Glob Public Health. 2026 May 8;6(5):e0006333. doi: 10.1371/journal.pgph.0006333 (PMC13155673; doi:10.1371/journal.pgph.0006333)
Supplement: S1 Text — (DOCX) [file pgph.0006333.s001.docx]

## **Operational Definitions**

1. *Pulmonary TB:* TB affecting the lung tissue. Any microbiological evidence of TB in the sputum or clinical TB in the lungs with or without the presence of the disease elsewhere is considered a case of pulmonary TB. Pulmonary TB marked in *Ni-kshay* will be taken as Pulmonary TB.
2. *Extra-pulmonary TB:* TB affecting any parts of the body other than the lungs is said to be extra-pulmonary TB. If a patient shows the presence of TB in the lungs along with sites other than the lungs, it will be considered a case of pulmonary TB. Extra-pulmonary TB marked in *Ni-kshay* will be taken as Extra-Pulmonary TB.
3. *Drug-resistant TB (DR-TB):* Microbiologically confirmed TB patients with evidence of resistance to one or more conventionally used anti-TB drugs. In this paper, DR-TB is used for TB patients marked as DRTB in the *Ni-kshay* notification register.
4. *Private Provider/Private Health Care provider***:** Any medical practitioner treating TB cases using modern medicine and is marked as a private provider/facility in *Ni-kshay* will be considered a private provider.
5. *Patient Provider Support Agency (PPSA):* It is an agency/NGO/service provider contracted by the state/district for a fixed contract period to offer a bundle of services to strengthen engagement with private sector providers. It provides a range of services - mapping and landscaping of private providers, enrolment of presumptive TB patients, case notification, linkage to free diagnostics and treatment, patient counseling and adherence support, comorbidity screening, universal drug susceptibility testing, follow-up and reporting treatment outcomes, and contact investigation, varying district to district based on contract terms.
6. *Operational Patient Provider Support Agency (PPSA) or PPSA Initiation-* PPSA will be considered operational from the date/month of contract initiation as per MoU with the State/districts**.**
7. *Linked with Programmatic FDC:* Notified private sector TB patients who were linked to and provided programmatic fixed drug combination (FDC) while still on consultation/treatment with a private doctor.
8. *Nikshay Poshan Yojana (NPY) and linkage to NPY*: NTEP offers a direct benefit transfer incentive to support the nutrition of TB patients (Rs. 1000 per month for the entire treatment duration). Every notified TB patient (both public and private) is eligible for this benefit. After notification, patient’s bank account details are to be collected and entered in *Ni-kshay* to link them for availing NPY benefits (known as linkage).
9. *Pretreatment loss to follow up*: Gap of number of notified TB cases and number of TB cases initiated on treatment as per *Ni-ksha*y in within a set timeframe for a district/state will be counted as pretreatment loss to follow up.
10. *Pre-treatment delay*: The NTEP program focuses on the start of TB treatment as early as possible after notification (preferably immediately). A delay of more than 7 days between the date of diagnosis and the date of treatment initiation has been considered as pre-treatment delay for the study.
11. *TB care services*: A Set of services which are to be offered to notified TB cases as part of public health action till treatment completion as per program guidelines. For the study TB care services-linkage to fixed drug combination (FDC) under the program, co-morbidity testing (HIV and diabetes mellitus), Nucleic Acid Amplification test (NAAT) for drug susceptibility testing (DST) for rifampicin, and linkage with *Ni-kshay* Poshan Yojana through bank seeding are considered.
12. *Nucleic Acid Amplification Technology (NAAT*): Molecular Cartridge-Based Nucleic Acid Amplification Technology using Computerized GeneXpert/Truenat Machines. This technology detects Mycobacterium TB along with the rifampicin resistance status.
13. *Valid Drug susceptibility test:* Results from WHO recommended rapid molecular test indicating susceptibility as resistance detected or resistance not detected. It excluded all other results such as error, invalid and indeterminate.
14. *Microbiologically confirmed TB:* TB diagnosed in a biological specimen by smear microscopy, culture, or a WHO-endorsed rapid molecular test adopted by NTEP
15. *Upfront NAAT:* When NAAT test is offered as first test to diagnose presumptive TB sample, it is termed upfront NAAT, which detect TB mycobacteria as well as susceptibility to TB drugs.
