# Supplementary material for: Enhancing tuberculosis care in the private sector: Role of innovative private sector engagement model under programmatic settings in India
Source: PLOS Glob Public Health. 2026 May 8;6(5):e0006333. doi: 10.1371/journal.pgph.0006333 (PMC13155673; doi:10.1371/journal.pgph.0006333)
Supplement: S1 Table — (DOCX) [file pgph.0006333.s002.docx]

## **List of states and districts as per National Tuberculosis Elimination Programme in India with PPSA operational in 2023 (Included in the study)**

| **Table: State wise list of districts supported with PPSA throughout the year in 2023 (n=204), included in study** | | |
| --- | --- | --- |
| **S.No** | **States** | **Districts** |
| 1 | Andhra Pradesh | Alluri Sitharama Raju, Anakapalli, Ananthapuramu, Annamayya, Bapatla, Dr B R Ambedkar Konaseema, East Godavari, Guntur, Kakinada, Krishna, NTR, Palnadu, Parvathipuram Manyam, Prakasam, Sri Potti Sriramulu Nellore, Sri Sathya Sai, Srikakulam, Visakhapatnam, Vizianagaram, Y.S.R |
| 2 | Assam | Barpeta, Cachar, Charaideo, Dibrugarh, Goalpara, Hojai, Jorhat, Kamrup Metro, Majuli, Nagaon, Sibsagar, Sonitpur, Tinsukia |
| 3 | Bihar | Bhojpur, Darbhanga, Gopalganj, Madhubani, Muzaffarpur, Nalanda, Pashchim Champaran, Patna, Purba Champaran, Sitamarhi, Siwan |
| 4 | Chhattisgarh | Bilaspur, Dhamtari, Durg, Korba, Raigarh, Raipur |
| 5 | Gujarat | Arvalli, Banaskantha, Bhavnagar, Bhavnagar Municipal Corporation, Gandhinagar, Gandhinagar Municipal Corporation, Jamnagar Municipal Corporation, Jamnagar-Rural, Junagadh, Junagadh Municipal Corporation, Mahesana, Morbi, Patan, Rajkot, Rajkot Municipal Corporation, Sabarkantha, Surendranagar |
| 6 | Jharkhand | Bokaro, Chatra, Garhwa, Giridih, Gumla, Hazaribagh, Khunti, Kodarma, Latehar, Lohardaga, Palamu, Pashchimi Singhbhum, Purbi Singhbhum, Ramgarh, Ranchi, Saraikela-Kharsawan, Simdega |
| 7 | Madhya Pradesh | Anuppur, Ashoknagar, Balaghat, Bhind, Chhatarpur, Chhindwara, Damoh, Guna, Gwalior, Jabalpur, Katni, Mandla, Morena, Narsinghpur, Niwari, Panna, Rewa, Sagar, Satna, Seoni, Shahdol, Sheopur, Shivpuri, Sidhi, Singrauli, Tikamgarh, Umaria |
| 8 | Maharashtra | Akola Muncipal Corporation, Amravati Muncipal Corporation, Chandrapur, Dhule Muncipal Corporation, Jalgaon Muncipal Corporation, Latur, Mumbai-Andheri East, Mumbai-Andheri West, Mumbai-Bail Bazar Road, Mumbai-Bandra East, Mumbai-Bandra West, Mumbai-Borivali, Mumbai-Byculla, Mumbai-Centenary, Mumbai-Chembur, Mumbai-Colaba, Mumbai-Dadar, Mumbai-Dahisar, Mumbai-Ghatkopar, Mumbai-Goregaon, Mumbai-Govandi, Mumbai-Grant Road, Mumbai-Kandivali, Mumbai-Kurla, Mumbai-Malad, Mumbai-Mulund, Mumbai-Parel, Mumbai-Prabhadevi, Mumbai-Sion, Mumbai-Vikhroli, Nagpur Muncipal Corporation, Nanded Waghala Muncipal Corporation, Nandurbar, Parbhani, Pune Rural |
| 9 | Manipur | Imphal East, Imphal West |
| 10 | Mizoram | Aizawl |
| 11 | Odisha | Bhubaneshwar Mc, Cuttack, Ganjam, Khordha, Mayurbhanj, Sambalpur, Sundargarh |
| 12 | Punjab | Amritsar, Patiala |
| 13 | Telangana | Hyderabad, Jagtial, Karimnagar, Medchal Malkajgiri, Nizamabad, Rangareddy, Sangareddy, Siddipet, Suryapet, Vikarabad |
| 14 | Uttar Pradesh | Agra, Aligarh, Ayodhya, Azamgarh, Bahraich, Ballia, Banda, Barabanki, Bareilly, Basti, Bijnor, Bulandshahar, Firozabad, Gautam Budh Nagar, Ghaziabad, Gonda, Gorakhpur, Hardoi, Jaunpur, Jhansi, Kanpur Nagar, Kheri, Lalitpur, Lucknow, Mathura, Meerut, Mirzapur, Moradabad, Muzaffarnagar, Prayagraj, Rae Bareli, Rampur, Saharanpur, Shahjahanpur, Sitapur, Varanasi |
