# Supplementary material for: Enhancing tuberculosis care in the private sector: Role of innovative private sector engagement model under programmatic settings in India
Source: PLOS Glob Public Health. 2026 May 8;6(5):e0006333. doi: 10.1371/journal.pgph.0006333 (PMC13155673; doi:10.1371/journal.pgph.0006333)
Supplement: S2 Table — (DOCX) [file pgph.0006333.s003.docx]

## **List of states and districts as per National Tuberculosis Elimination Programme in India without PPSA operation in 2023 (Included in the study)**

| **Table : State wise list of districts without PPSA support throughout the year in 2023 (n=529), included in study** | | |
| --- | --- | --- |
| **S.No** | **State** | **Districts** |
| 1 | Andaman & Nicobar Islands | Andamans & Nicobars, North & Middle Andaman, South Andaman |
| 2 | Arunachal Pradesh | Changlang, East Siang, Lohit, Papumpare, Tirap |
| 3 | Assam | Baksa, Biswanath, Chirang, Dima Hasao, Hailakandi, Karbi Anglong, Marigaon, South Salmara Mankachar, Udalguri, West Karbi Anglong |
| 4 | Bihar | Araria, Arwal, Aurangabad-Bi, Banka, Buxar, Jamui, Jehanabad, Kaimur, Khagaria, Kishanganj, Lakhisarai, Madhepura, Nawada, Purnia, Rohtas, Saran, Sheikhpura, Sheohar, Supaul |
| 5 | CHANDIGARH | Chandigarh |
| 6 | Chhattisgarh | Balod, Baloda Bazar, Balrampur, Bastar, Bemetara, Bijapur, Dantewada, Gariyaband, Janjgir-Champa, Jashpur, Kabirdham, Kanker, Kondagaon, Koriya, Mahasamund, Manendragarh-Chirmiri-Bharatpur, Mohla-Manpur-Ambagarhchowki, Mungeli, Narayanpur, Rajnandgaon, Sakti, Sarangarh Bilaigarh, Sarguja, Sukma, Surajpur |
| 7 | Dadra and Nagar Haveli and Daman and Diu | Dadra & Nagar Haveli, Daman |
| 8 | Delhi | Bijwasan, BJRM Chest Clinic, BSA Chest Clinic, CD Chest Clinic, Chest Clinic Narela, Damien Foundation India Trust, DDU Chest Clinic, GTB Chest Clinic, Gulabi Bagh, Hedgewar Chest Clinic, Jhandewalan, Karawal Nagar, Kingsway, LN Chest Clinic, LRS, MNCH Chest Clinic, Moti Nagar, NDMC, Nehru Nagar, Patparganj, RK Mission, RTRM Chest Clinic, SGM Chest Clinic, Shahadra, SPM Marg, SPMH Chest Clinic |
| 9 | Gujarat | Ahmadabad Rural, Ahmedabad Municipal Corporation, Amreli, Anand, Bharuch, Botad, Chhotaudepur, Dahod, Devbhumi Dwarka, Gir Somnath, Kachchh, Kheda, Mahisagar, Narmada, Navsari, Panchmahals, Porbandar, Surat Municipal Corporation, Surat Rural, The Dangs, Vadodara Municipal Corporation, Vadodara Rural, Valsad, Vyara |
| 10 | Haryana | Ambala, Bhiwani, Charkhi Dadri, Faridabad, Fatehabad, Gurgaon, Hisar, Jhajjar, Jind, Kaithal, Karnal, Kurukshetra, Mahendragarh, Mewat, Palwal, Panchkula, Panipat, Rewari, Rohtak, Sirsa, Sonipat, Yamunanagar |
| 11 | Himachal Pradesh | Bilaspur-Hp, Chamba, Hamirpur-Hp, Kangra, Kullu, Lahul & Spiti, Mandi, Shimla, Sirmaur, Solan, Una-Hp |
| 12 | Jammu & Kashmir | Anantnag, Badgam, Baramulla, Doda, Jammu, Kathua, Kupwara, Poonch, Pulwama, Rajouri, Srinagar, Udhampur |
| 13 | Karnataka | Bagalkot, Belgaum, Bellary, Bengaluru City, Bengaluru Rural, Bengaluru Urban, Bidar, Chamarajanagar, Chikkaballapur, Chikmagalur, Chitradurga, Dakshina Kannada, Davanagere, Dharwad, Gadag, Hassan, Haveri, Kalaburagi, Kodagu, Kolar, Koppal, Mandya, Mysuru, Raichur, Ramanagara, Shimoga, Tumkur, Udupi, Uttara Kannada, Vijayanagara, Vijayapura, Yadgiri |
| 14 | Kerala | Alappuzha, Ernakulam, Idukki, Kannur, Kasaragod, Kollam, Kottayam, Kozhikode, Malappuram, Palakkad, Pathanamthitta, Thiruvananthapuram, Thrissur, Wayanad |
| 15 | Ladakh | Leh |
| 16 | Madhya Pradesh | Agar Malwa, Alirajpur, Barwani, Betul, Bhopal, Burhanpur, Datia, Dewas, Dhar, Dindori, Harda, Hoshangabad, Indore, Jhabua, Khandwa, Khargone, Mandsaur, Neemuch, Raisen, Rajgarh, Ratlam, Sehore, Shajapur, Ujjain, Vidisha |
| 17 | Maharashtra | Ahilyanagar, Ahilyanagar Muncipal Corporation, Akola, Amravati, Beed, Bhandara, Bhiwandi Nizampur Muncipal Corporation, Buldana, Chhatrapati Sambhajinagar, Chhatrapati Sambhajinagar Muncipal Corporation, Dharashiv, Dhule, Gadchiroli, Gondiya, Hingoli, Jalgaon, Jalna, Kalyan Dombivli Muncipal Corporation, Kolhapur, Kolhapur Muncipal Corporation, Malegaon Muncipal Corporation, Mira Bhayander Muncipal Corporation, Nagpur, Nanded, Nashik, Nashik Muncipal Corporation, Navi Mumbai Muncipal Corporation, Palghar, Pimpri Chinchwad Muncipal Corporation, Pune Muncipal Corporation, Raigad, Ratnagiri, Sangli, Sangli Muncipal Corporation, Satara, Sindhudurg, Solapur, Solapur Muncipal Corporation, Thane, Thane Muncipal Corporation, Ulhasnagar Muncipal Corporation, Vasai Virar Muncipal Corporation, Wardha, Washim, Yavatmal |
| 18 | Manipur | Bishnupur, Chandel, Churachandpur, Kakching, Tengnoupal, Thoubal, Ukhrul |
| 19 | Meghalaya | East Garo Hills, Ri Bhoi, West Garo Hills, West Khasi Hills |
| 20 | Mizoram | Lawngtlai |
| 21 | Nagaland | Dimapur, Kohima, Mokokchung, Phek, Tuensang, Wokha |
| 22 | Odisha | Anugul, Balangir, Baleshwar, Bargarh, Bhadrak, Boudh, Deogarh, Dhenkanal, Gajapati, Jagatsinghapur, Jajapur, Jharsuguda, Kalahandi, Kandhamal, Kendrapara, Kendujhar, Koraput, Malkangiri, Nabarangapur, Nayagarh, Nuapada, Puri, Rayagada, Sonapur |
| 23 | Puducherry | Puducherry |
| 24 | Punjab | Barnala, Bathinda, Faridkot, Fatehgarh Sahib, Fazilka, Firozpur, Gurdaspur, Hoshiarpur, Jalandhar, Kapurthala, Ludhiana, Mansa-Pn, Moga, Mohali, Muktsar, Nawanshahr, Pathankot, Rupnagar, Sangrur, Tarn Taran |
| 25 | Rajasthan | Ajmer, Alwar, Banswara, Baran, Barmer, Bharatpur, Bhilwara, Bikaner, Bundi, Chittaurgarh, Churu, Dausa, Dholpur, Dungarpur, Ganganagar, Hanumangarh, Jaipur I, Jaipur Ii, Jaisalmer, Jalore, Jhalawar, Jhunjhunun, Jodhpur, Karauli, Kota, Nagaur, Pali, Pratapgarh, Rajsamand, Sawai Madhopur, Sikar, Sirohi, Tonk, Udaipur |
| 26 | Sikkim | DTC Gangtok, Singtam, South District |
| 27 | Tamil Nadu | Central Chennai, Chennai, Coimbatore, Cuddalore, Dharmapuri, Dindigul, East Chennai, Erode, Kancheepuram, Kanniyakumari, Karur, Krishnagiri, Madurai, Nagapattinam, Namakkal, North Chennai, Perambalur, Pudukkottai, Ramanathapuram, Salem, Sivaganga, South Chennai, Thanjavur, The Nilgiris, Theni, Thiruvallur, Thiruvarur, Thoothukudi, Tiruchirappalli, Tirunelveli, Tiruppur, Tiruvannamalai, Vellore, Viluppuram, Virudhunagar, West Chennai |
| 28 | Telangana | Adilabad, Bhadradri Kothagudem, Hanumakonda, Jangaon, Jayashankar Bhupalpally, Jogulamba Gadwal, Kamareddy, Khammam, Kumurambheem Asifabad, Mahabubabad, Mahabubnagar, Mancherial, Medak, Mulugu, Nagarkurnool, Nalgonda, Narayanpet, Nirmal, Peddapalli, Rajanna Sircilla, Wanaparthy, Warangal, Yadadri Bhuvanagiri |
| 29 | Tripura | Dhalai, Khowai, North Tripura, Unakoti, West Tripura |
| 30 | Uttar Pradesh | Ambedkar Nagar, Amethi, Auraiya, Baghpat, Balrampur, Budaun, Chandauli, Chitrakoot, Deoria, Etah, Etawah, Farrukhabad, Fatehpur, Ghazipur, Hamirpur-Up, Hapur, Hathras, Jalaun, Jyotiba Phule Nagar, Kannauj, Kanpur Dehat, Kanshiram Nagar, Kaushambi, Kushinagar, Maharajganj, Mahoba, Mainpuri, Mau, Pilibhit, Pratapgarh, Sambhal, Sant Kabir Nagar, Sant Ravidas Nagar, Shamli, Shrawasti, Siddharthnagar, Sonbhadra, Sultanpur, Unnao |
| 31 | Uttarakhand | Almora, Bageshwar, Chamoli, Champawat, Dehradun, Haridwar, Nainital, Pauri Garhwal, Pithoragarh, Rudraprayag, Tehri Garhwal, Udhamsingh Nagar, Uttarkashi |
| 32 | West Bengal | Alipore (Kolkata), Alipurduar, Bagbazar (Kolkata), Bankura, Basirhat, Behala (Kolkata), Birbhum, Bishnupur, Cooch Behar, Dakshin Dinajpur, Darjiling, Diamond Harbour, Hazi (Kolkata), Hooghly, Howrah, Jalpaiguri, Jhargram, Kalimpong, Kolkata (Non Functional District), Malda, Maniktala (Kolkata), Manshatala (Kolkata), MTMTB (Kolkata), Murshidabad, Nadia, Nandigram Hd, North 24 Parganas, Paschim Bardhaman, Paschim Medinipur, Purba Bardhaman, Purba Medinipur, Purulia, Rampurhat, South 24 Parganas, Strand Bank (Kolkata), Tangra (Kolkata), Tollygunge (Kolkata), Uttar Dinajpur |
