# Supplementary material for: Enhancing tuberculosis care in the private sector: Role of innovative private sector engagement model under programmatic settings in India
Source: PLOS Glob Public Health. 2026 May 8;6(5):e0006333. doi: 10.1371/journal.pgph.0006333 (PMC13155673; doi:10.1371/journal.pgph.0006333)
Supplement: S3 Table — (DOCX) [file pgph.0006333.s004.docx]

## **List of states and districts as per National Tuberculosis Elimination Programme in India excluded from the study**

| **Table 3: State wise list of districts supported with PPSA for less than the entire year (partial presence) in 2023 (n=35), excluded from study** | | |
| --- | --- | --- |
| **S.No** | **States** | **Districts** |
| 1 | Andhra Pradesh | Chittoor, Eluru, Kurnool, Nandyal, Tirupati, West Godavari |
| 2 | Assam | Bongaigaon, Darrang, Dhemaji, Dhubri, Golaghat, Kamrup, Karimganj, Kokrajhar, Lakhimpur, Nalbari |
| 3 | Bihar | Begusarai, Bhagalpur, Gaya, Katihar, Munger, Saharsa, Samastipur, Vaishali |
| 4 | Goa | North Goa, South Goa |
| 5 | Jharkhand | Deoghar, Dhanbad, Dumka, Godda, Jamtara, Pakur, Sahibganj |
| 6 | Meghalaya | East Khasi Hills, Jaintia Hills |
